# Awareness of Myocardial Infarction Symptoms and Risk Factors in Saudi Arabia: A Cross-Sectional Study

**DOI:** 10.7759/cureus.50092

**Published:** 2023-12-07

**Authors:** Saad M Alsaab, Ahmed M Almutairi, Ghadi K Alsaadi, Ziyad A Altokhais, Samar H Alabdulqader, Wafa Y Alnofal, Nourah M Alduhaim

**Affiliations:** 1 Department of Internal Medicine, College of Medicine, Shaqra University, Riyadh, SAU; 2 College of Medicine, University of Jeddah, Jeddah, SAU; 3 College of Medicine, Taif University, Taif, SAU; 4 College of Medicine, Shaqra University, Dawadmi, SAU; 5 College of Medicine, King Faisal University, Al-Ahsa, SAU; 6 Department of Internal Medicine, King Saud Medical City, Riyadh, SAU; 7 Department of Medicine, AlQuwayiyah General Hospital, Riyadh, SAU

**Keywords:** saudi arabia, middle east, myocardial infarction, cardiovascular disease, awareness

## Abstract

Background

Cardiovascular disease is the leading global cause of mortality. Recognition of myocardial infarction (MI) symptoms is crucial as it influences clinical outcomes. Furthermore, awareness of MI risk factors assists in obtaining healthier lifestyles, alleviating its burden and mortality rates. This study aims to evaluate the awareness levels of the general population in Saudi Arabia regarding MI symptoms and risk factors and to identify its determinants.

Methods

A descriptive cross-sectional study was conducted among the general Saudi population, with 1,247 participants, excluding those residing outside Saudi Arabia and healthcare-related individuals. An online self-administered questionnaire encompassed sociodemographic characteristics, awareness of MI symptoms and risk factors, and participants' perceptions concerning appropriate responses during an MI event.

Results

The majority of participants recognized chest pain and shortness of breath as MI symptoms, with a percentage of 87.1% and 86%, respectively. Risk factors awareness was substantial at 90.3% for smoking and 76.1% for obesity. The mean overall awareness score was 26.6±7.5, and around 36% were aware of both symptoms and risk factors. Higher education levels were positively associated with both risk factors and symptom awareness, while employment status showed negative associations with both. Higher-income correlated with greater risk factors awareness. Gender remained significant, with males exhibiting a lower awareness of risk factors and symptoms. Region and chronic disease status were positively associated with risk factors and symptom awareness. More than 90% of participants perceived going to the emergency room immediately if they recognized an MI attack.

Conclusion

This study highlights the necessity for inclusive awareness campaigns to enhance the identification of MI symptoms and risk factors in Saudi Arabia. It should focus on groups with limited awareness, such as males, employed and retired individuals, and specific pronounced regional disparities.

## Introduction

Myocardial infarction (MI) is defined as myocardial cell necrosis resulting from oxygen deprivation secondary to complete or decreased flow of blood to a specific area of the myocardium. This event might pass unnoticed or result in a catastrophic hemodynamic deterioration leading to death [1].

Cardiovascular diseases (CVDs) account for a majority of deaths [2]. According to the World Health Organization (WHO), in 2019, CVDs were the leading cause of death worldwide, accounting for 17.9 million deaths worldwide each year [2]. According to the American Heart Association Statistics Committee and Stroke Statistics Subcommittee, in 2006, out of a total number of 71,300,000 study subjects, coronary artery disease (CAD) accounted for a total of 13,200,000, of which MI represented 7,200,000 of the cases [3].

Saudi Arabia has experienced tremendous urbanization in recent years, which has increased CVD rates, with an overall incidence of 5.5% [4]. The Saudi Acute MI Registry (STARS), which was managed under the supervision of the Saudi Heart Association, intended to overcome the previous studies' limitations with the first representative survey of the registry program (STARS-1 Program) measuring long-term outcomes and provided care. The overall mortality was 4%, 5.8%, and 8.1%, respectively, in hospital, at one month, and at one-year follow-up intervals [5].

Presentation of ischemic pain in MI atypically could present as epigastric discomfort at rest or exertion [6]. The main observed symptom is pressure such as retrosternal chest pain or heaviness radiating to the neck and arms, more commonly the left shoulder. Pain is usually not triggered by specific factors and can be continuous or intermittent, but it usually persists for 20 minutes or less for a single attack [7]. Other symptoms may include nausea, diaphoresis, dyspnea, abdominal pain, syncope, and fatigue [6,8,9]. MI presentation could range from atypical subtle symptoms such as palpitations to cardiac arrest, and, in some cases, it may occur silently [10].

The major outlined risk factors included a sedentary lifestyle, hypertension, diabetes, and moderate alcohol consumption, which were related to a higher MI incidence in female rather than male participants. Meanwhile, the remaining risk factors of deranged lipid profile, smoking, central obesity, stress, and unhealthy diet were associated with a similar rate of MI in both genders [11].

It should be emphasized that the Middle Eastern region, including Saudi Arabia, has a disproportionately high burden of CAD risk factors. It was also noted that patients had at least three risk factors for CAD. Hypertension, diabetes, and smoking were present in around half of the patients, and dyslipidemia was present in at least one-third of the patients. Furthermore, the vast majority of those individuals had at least one uncontrolled risk factor, indicating a lack of disease knowledge [12].

With the increasing incidence of MI, our study was conducted to measure the knowledge of our society about risk factors and presentation, involving all four main regions of Saudi Arabia. In addition, we included a tool in our survey to explore the competency of the population in handling such events. Identifying heart attack warning signals and risk factors may influence an individual's, family's, or bystander's decision time in seeking medical attention. The intent of this paper is to improve the awareness of MI among non-health-related individuals and hopefully the control and prevention of it as a result.

## Materials and methods

This is a descriptive cross-sectional study conducted among the general population of Saudi Arabia, which is estimated to be 36 million, to assess the level of awareness of MI symptoms and risk factors in our society, in addition to the proper response in the event of MI. We included individuals between 18 and 75 years of age, excluding those who reside outside Saudi Arabia. The sample size was calculated using Epi Info software, considering a 95% confidence level, 50% anticipated population proportion, 5% margin of error, and a population of 36 million; the minimum sample size was 384 participants. Data collection was commenced in April 2023 and completed by August 2023, with a final sample size of 1,247 participants.

A preliminary pilot study involving a selected group of participants was conducted to assess the questionnaire's validity and understandability. Participant feedback from this preliminary phase was used to improve the final survey (available in the Appendices). Utilizing convenient sampling, an online self-administrated questionnaire created using Google Forms, available in Arabic and English, was disseminated to participants through media platforms (WhatsApp, Instagram, Twitter, and Facebook); in regions that lacked adequate responses, manual survey distribution was done with the assistance of data collectors. Individuals were invited to complete the questionnaire voluntarily by either accessing the Google form link or by completing a hard copy of the questionnaire, which included an agreement to participate section/page, with participation being anonymous to ensure the confidentiality of participants.

The questionnaire was divided into three main sections. The first section collected demographic data encompassing age, gender, nationality, marital status, employment status, educational attainment, monthly income, and any chronic diseases the participants had. The second section featured a questionnaire derived from previous studies [13]. It explored the participant's knowledge regarding symptoms and risk factors of MI. The third section evaluated participants' perceptions regarding appropriate responses in cases of an MI event.

For the primary assessment of MI awareness, a structured scoring system was implemented. Participants whose responses were "likely" received a score of 2, while those whose responses were "unlikely" were assigned a score of 0. Neutral responses were allocated a score of 1. Subsequently, a cumulative awareness score was computed for each participant, resulting in scores within a range of 0 to 40 points. Higher scores denoted greater levels of awareness.

To categorize overall awareness level, participants who scored 75% or more out of 40 correct answers "likely" were classified as aware. The awareness of each subscale was computed using the same method. Continuous variables were summarized using the mean and standard deviation, while categorical variables were summarized using frequencies and percentages. To identify factors associated with MI awareness, multiple linear regression analyses were performed, with a statistical significance set at a p-value of less than 0.05.

Ethical approval

Ethical approval was obtained from The Local Committee at Shaqra University (HAPO-01-R-128) with approval number ERC_SU_20230041.

## Results

Excluding individuals residing outside of Saudi Arabia and healthcare-related individuals, 1,247 participants were included in the study. Most of these participants fell within the age group of 18 to 39 years, constituting 56.5% of the cohort. Regarding gender distribution, females comprised the larger portion at 67.8%, and the majority were Saudi citizens (97.4%). A significant portion of the participants were married (57.1%), held bachelor's degrees (69.2%), and were residents of the central region of Saudi Arabia (45.7%). Most participants reported employment (34.6%) and indicated a monthly income ranging from 5,000 to 15,000 Saudi Riyal (36.4%). Notably, 71.7% stated the absence of chronic diseases, while 11.1% confirmed a diagnosis of diabetes mellitus (Table 1).

**Table 1:** Sociodemographic characteristics of the study participants. n, number of participants; %, percentage; SR, Saudi Riyal

Characteristic	n (%)
Age (years)	
18–39	704 (56.5)
40–61	482 (38.7)
More than 61	61 (4.9)
Gender	
Female	846 (67.8)
Male	401 (32.2)
Nationality	
Non-Saudi	33 (2.6)
Saudi	1,214 (97.4)
Marital status	
Single	468 (37.5)
Married	712 (57.1)
Divorced	42 (3.4)
Widowed	25 (2.0)
Level of education	
Primary	38 (3.1)
Secondary	254 (20.4)
Bachelor	861 (69.2)
Higher education	92 (7.4)
Employment status	
Student	329 (26.4)
Employed	432 (34.6)
Freelancer	65 (5.2)
Unemployed	237 (19.0)
Retired	184 (14.8)
Monthly income	
<5,000 SR	560 (44.9)
5,000–15,000 SR	454 (36.4)
15,000–40,000 SR	203 (16.3)
>40,000 SR	30 (2.4)
Residence	
North	92 (7.4)
South	141 (11.3)
East	161 (12.9)
West	283 (22.7)
Center	570 (45.7)
What chronic diseases do you have?	
Hypertension	120 (9.6)
Diabetes mellitus	139 (11.1)
Dyslipidemia	131 (10.5)
Chronic kidney disease	3 (0.2)
Others	100 (8.0)
None	894 (71.7)

Recognizing the symptoms associated with MI, 87.1% of the participants identified chest pain or discomfort, followed by shortness of breath at 86% and palpitations at 67.1%. Regarding risk factors, a considerable percentage (90.3%) recognized smoking as a risk factor for MI, while 76.1% identified obesity and 72.9% identified hypertension as an associated risk factor.

**Figure 1:**
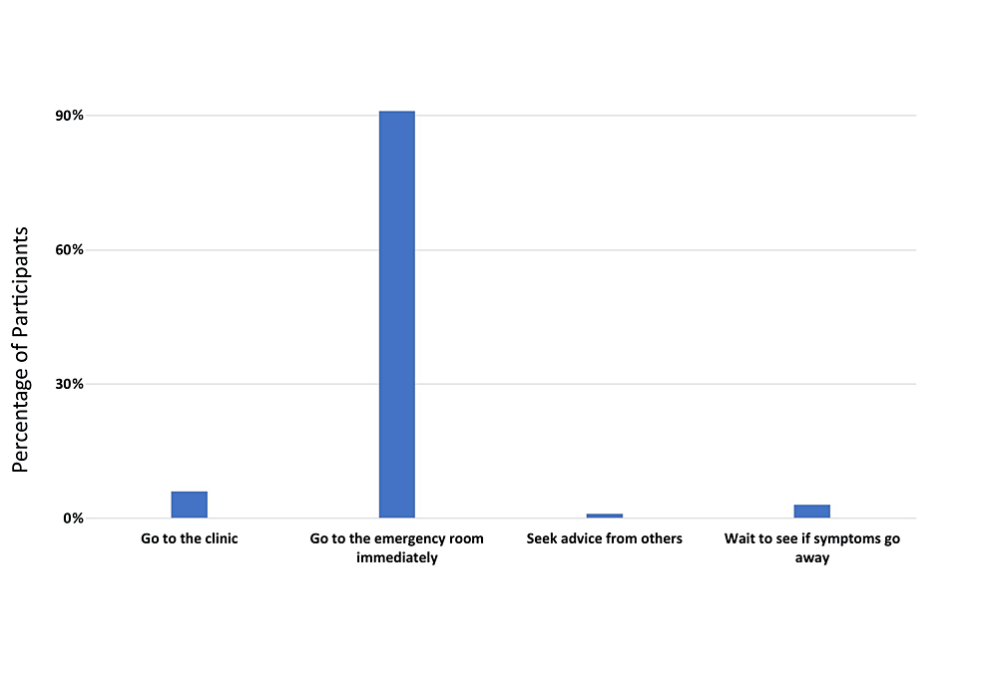
Participants’ perception regarding appropriate response in case of recognizing a myocardial infarction event.

The mean overall score was 26.6±7.5, with a median of 27 and a range of 0 to 40. Approximately 36% demonstrated awareness of both MI symptoms and risk factors. The mean risk factor score was 13.7±4.4 with a median of 14, while the mean symptoms score was 12.9±4.1 with a median of 13. These subscales also had a range of 0 to 20. Of the participants, roughly 44.3% were aware of MI risk factors, whereas 34% were aware of MI symptoms. More than 90% of participants perceived going to the emergency room immediately if they encountered an MI event (Table 2, Figure 1).

**Table 2:** Respondents’ awareness about myocardial infarction risk factors and symptoms. n, number of respondents; %, percentage

Characteristic	Likely, n (%)	Unlikely, n (%)	Not sure, n (%)
Symptoms of myocardial infarction			
Pain or discomfort in the chest	1,086 (87.1)	62 (5.0)	99 (7.9)
Shortness of breath	1,073 (86)	94 (7.5)	80 (6.4)
Sweating	779 (62.5)	220 (17.6)	248 (19.9)
Nausea	504 (40.4)	354 (28.4)	389 (31.2)
Dizziness	653 (52.4)	283 (22.7)	311 (24.9)
Pain in the upper part of the abdomen	503 (40.3)	359 (28.8)	385 (30.9)
Palpitation	837 (67.1)	208 (16.7)	202 (16.2)
Heartburn	291 (23.3)	509 (40.8)	447 (35.8)
Vomiting	346 (27.7)	461 (37)	440 (35.3)
Back pain	458 (36.7)	370 (29.7)	419 (33.6)
Risk factors of myocardial infarction			
Smoking	1,126 (90.3)	60 (4.8)	61 (4.9)
Hypertension	909 (72.9)	167 (13.4)	171 (13.7)
Diabetes mellitus	569 (45.6)	341 (27.3)	337 (27.1)
High level of cholesterol	853 (68.4)	189 (15.2)	205 (16.4)
Family history of heart attack	861 (69)	176 (14.2)	210 (16.8)
Obesity	949 (76.1)	160 (12.8)	138 (11.1)
Age	668 (53.6)	335 (26.9)	244 (19.6)
Gender	289 (23.2)	571 (45.8)	387 (31)
Sleep disturbance	421 (33.8)	459 (36.8)	367 (29.4)
Unhealthy diet	729 (58.5)	263 (21.1)	255 (20.4)

Factors influencing awareness of MI were investigated. Results indicated that males exhibited lower awareness of risk factors than females (95% CI: -1.1, -0.06, p-value: 0.031). Furthermore, participants with a bachelor's degree (95% CI: 0.45, 3.2, p-value: 0.009) or higher education (95% CI: 0.34, 3.6, p-value: 0.018) displayed greater risk factor awareness than those with primary education.

Employment status played a role as well, with employed individuals (95% CI: -2.1, -0.45, p-value: 0.003), freelancers (95% CI: -2.9, -0.54, p-value: 0.004), unemployed (95% CI: -2.2, -0.74, p-value:<0.001), and retirees (95% CI: -2.4, -0.40, p-value: 0.006) demonstrating lower risk factor awareness compared to students. Higher-income levels were associated with heightened risk factors awareness compared to those with incomes below 5,000 SR (95% CI: 0.09, 1.6, p-value: 0.029). Geographically, residents outside the central region exhibited greater awareness, and those with chronic diseases displayed superior awareness (95% CI: 0.41, 1.5, p-value: <0.001) (Table 3).

**Table 3:** Predictors of myocardial infarction risk factors awareness among the general population of Saudi Arabia. CI, confidence interval; SR, Saudi Riyal

Characteristics	Beta	95% CI	p-value
Gender			
Female	–	–	–
Male	-0.60	-1.1, -0.06	0.031
Level of education			
Primary	–	–	–
Secondary	1.3	-0.17, 2.7	0.084
Bachelor	1.8	0.45, 3.2	0.009
Higher education	2.0	0.34, 3.6	0.018
Employment status			
Student	–	–	–
Employed	-1.3	-2.1, -0.45	0.003
Freelancer	-1.7	-2.9, -0.54	0.004
Unemployed	-1.5	-2.2, -0.74	<0.001
Retired	-1.4	-2.4, -0.40	0.006
Monthly income			
<5,000 SR	–	–	–
5,000–15,000 SR	0.82	0.09, 1.6	0.029
15000–40,000 SR	0.74	-0.14, 1.6	0.10
>40,000 SR	0.42	-1.2, 2.1	0.6
Residence			
Center	–	–	–
North	4.5	3.5, 5.4	<0.001
South	1.5	0.70, 2.4	<0.001
East	0.94	0.20, 1.7	0.013
West	1.8	1.2, 2.5	<0.001
Do you have any chronic diseases?			
No	–	–	–
Yes	0.97	0.41, 1.5	<0.001

Regarding symptom awareness, males displayed lower awareness than females (95% CI: -1.6, -0.55, p-value: <0.001). Individuals with secondary education (95% CI: 0.27, 3.0, p-value: 0.019), bachelor's degrees (95% CI: 1.0, 3.7, p-value: <0.001), or higher education (95% CI: 0.73, 3.9, p-value: 0.004) exhibited greater awareness compared to those with primary education. Employment status again influenced awareness, as those employed (95% CI: -1.8, -0.52, p-value: <0.001), freelancers (95% CI: -2.8, -0.59, p-value: 0.003), unemployed (95% CI: -2.0, -0.54, p-value: <0.001), and retired individuals (95% CI: -2.3, -0.64, p-value: <0.001) demonstrated lower awareness relative to students. Additionally, residents in the north (95% CI: 0.51, 2.3, p-value: 0.0025) and west regions (95% CI: 0.77, 2.0, p-value: <0.001) showed greater awareness compared to their central region counterparts. Furthermore, individuals with chronic diseases showed heightened awareness (95% CI: 0.53, 1.6, p-value: <0.001) (Table 4).

**Table 4:** Predictors of myocardial infarction symptom awareness among the general population of Saudi Arabia. CI, confidence interval; SR, Saudi Riyal

Characteristic	Beta	95% CI	p-value
Gender			
Female	–	–	–
Male	-1.1	-1.6, -0.55	<0.001
Level of education			
Primary	–	–	–
Secondary	1.6	0.27, 3.0	0.019
Bachelor	2.4	1.0, 3.7	<0.001
Higher education	2.3	0.73, 3.9	0.004
Employment status			
Student	–	–	–
Employed	-1.2	-1.8, -0.52	<0.001
Freelancer	-1.7	-2.8, -0.59	0.003
Unemployed	-1.3	-2.0, -0.54	<0.001
Retired	-1.5	-2.3, -0.64	<0.001
Monthly income			
<5,000 SR	–	–	–
5,000–15,000 SR	0.29	-0.41, 0.99	0.4
15,000–40,000 SR	0.04	-0.80, 0.88	>0.9
>40,000 SR	0.77	-0.82, 2.4	0.3
Residence			
Center	–	–	–
North	1.4	0.51, 2.3	0.002
South	0.32	-0.49, 1.1	0.4
East	0.03	-0.68, 0.74	>0.9
West	1.4	0.77, 2.0	<0.001
Do you have any chronic diseases?			
No	–	–	–
Yes	1.1	0.53, 1.6	<0.001

## Discussion

The present study aimed to assess awareness of MI symptoms and risk factors among individuals residing in Saudi Arabia. The findings revealed that most participants were female, were Saudi citizens, were married, held bachelor's degrees, and resided in the central region of Saudi Arabia. These demographic characteristics are compatible with previous studies conducted in the United States [14].

Regarding awareness of MI symptoms, the participants demonstrated a high level of perception of chest pain or discomfort, shortness of breath, and palpitations, consistent with previous research indicating that chest pain is the most commonly recognized symptom of MI [15]. Likewise, a significant percentage of participants recognized smoking, obesity, and hypertension as risk factors for MI. Our study reported that 90.3% of participants identified smoking as a risk factor, while Basham et al. found that 85% of participants were aware of this association; however, the percentage difference could be attributed to the fact that the study of Basham et al. had a sample size of 1,689 participants [16].

Similar to the findings reported by Park et al., who found a moderate level of awareness among their participants [15], the mean score for symptom awareness in our study was 12.9±4.1, indicating a moderate level of awareness among our participants as well. However, in comparison to the findings of Khalifa et al., who reported a higher percentage of participants being aware of MI risk factors, it is important to note that the present study used a different methodology to assess risk factors awareness, which might have predisposed for the trivial variation in results. Additionally, the present study included participants from various regions of Saudi Arabia, while the study conducted by Khalifa et al. was narrowed to Riyadh City. Therefore, the differences in methodology and sample population may contribute to the variations in results between the two studies [17].

Parameters influencing awareness of MI symptoms and risk factors were further investigated. Participants with higher education levels, such as a bachelor's degree or higher, displayed greater risk factor awareness than those with primary education; this supports previous research findings constituting a positive association between education level and awareness of CVDs [14,15]. The difference in education level may be attributed to varying access to educational opportunities and resources, which can impact individuals' knowledge and understanding of health-related topics. The findings emphasize the effect of education in the prevention of MI and the importance of addressing society-centered health education campaigns.

It was also found that males exhibited lower awareness of risk factors than females, which is consistent with previous studies that reported gender differences in awareness of CVDs [13]. This difference in gender distribution may be due to cultural or societal factors specific to Saudi Arabia, which could influence the level of awareness among different genders. Previous research showed a positive association between education level and awareness of CVDs [13]. Similarly, in our study, participants with higher education levels, such as a bachelor's degree or higher, displayed greater risk factor awareness than those with primary education.

Geographically, residents outside the central region exhibited greater awareness; this difference in geographic areas could be attributed to the variations in healthcare access and resources across the different regions of Saudi Arabia. In contrast, a study performed in Jeddah by Al-Orainan et al. found that residents in urban areas had higher awareness levels compared to residents of rural areas; the disparity might be related to the different assessment tools used in the research; however, further studies are needed to explore these findings [18]. Moreover, individuals residing in areas with less residential population, better healthcare infrastructure, and services may have more opportunities for health education and awareness campaigns, leading to higher knowledge levels of MI. Therefore, educational efforts should be directed at the areas with less medical access.

Additionally, individuals with chronic diseases displayed elevated awareness, which suggests that individuals with existing health conditions may be more knowledgeable about risk factors and symptoms of CVDs.

Compared to the study conducted by Park et al. [15], our study found similar trends in terms of gender distribution, with females comprising the larger portion of participants. However, our study included a larger sample size and provided more detailed information on various demographic and socioeconomic factors influencing awareness of MI symptoms and risk factors. Moreover, our study highlighted the need for targeted interventions based on specific demographic characteristics, such as education level, employment status, and geographic location.

Our paper found that individuals with chronic diseases displayed greater awareness of MI symptoms and risk factors, suggesting that individuals with existing health conditions may be more informed about CVDs due to their experiences and interactions with healthcare professionals.

The findings propose a need for targeted educational interventions to improve awareness, particularly among certain demographic groups, such as male individuals with lower education.

The limitations of this study include sample bias due to the inclusion of only participants from Saudi Arabia, which may limit the generalizability of the study, reliance on self-reported data, which may be subject to recall or social desirability bias, the use of a cross-sectional design limiting the ability to establish causal relationships, and non-probability sampling, which may introduce selection bias.

## Conclusions

This study highlights the necessity for inclusive awareness campaigns to enhance the identification of MI symptoms and risk factors in Saudi Arabia. The findings of this study propose a need for targeted educational interventions to improve awareness, particularly among certain groups with limited awareness, such as males with lower education, retired individuals, and specific pronounced regional disparities.

## Appendices

**Table 5:** Questionnaire SR, Saudi Riyal

1	Age:	18–28						29–39							40–50					51–61						62–75						>75
2	Gender:											Male													Female							
3	Nationality:											Saudi													Non-Saudi							
4	Marital status:					Single								Married								Divorced							Widowed			
5	Level of education:	Illiterate						Primary							Secondary					High						Bachelor						Higher Education
6	Employment status:		Student							Employed							Unemployed							Freelancer							Retired	
7	Monthly income:				<5,000 SR									5,000–15,000 SR							15,000–40,000 SR							>40,000 SR				
8	Place of residence (e.g. Riyadh, Makkah, Dammam…):																															
9	Chronic diseases:	Hypertension					Diabetes mellitus						Dyslipidemia						Chronic kidney disease						None					Other (please write)		
10	Have you heard the term heart attack before?								Yes														No									
11	From the symptoms below, please select which you consider a symptom of heart attack (please select either likely, unlikely, or not sure for each answer):																															
Symptoms					1=Likely											2=Unlikely											3=Not sure					
Pain or discomfort in the chest																																
Shortness of breath																																
Sweating																																
Nausea																																
Dizziness																																
Pain in the upper part of the abdomen																																
Palpitation																																
Heart burn																																
Vomiting																																
Back pain																																
12	From the risk factors below, please select which you consider a risk factor of heart attack (please select either likely, unlikely, or not sure for each answer):																															
Risk factor					1=Likely											2=Unlikely											3=Not sure					
Smoking																																
Hypertension																																
Diabetes mellitus																																
High level of cholesterol																																
Family history of heart attack																																
Obesity																																
Age																																
Gender																																
Sleep disturbance																																
Unhealthy diet																																
13	What is the right action to do if you think that you have symptoms of heart attack?			Go to the emergency room immediately							Go to primary health care clinic							Wait to see if symptoms resolve											Seek advice from others			
